# *Gardenia jasminoides* J. Ellis extract GJ-4 attenuates hyperlipidemic vascular dementia in rats via regulating PPAR-γ-mediated microglial polarization

**DOI:** 10.29219/fnr.v66.8101

**Published:** 2022-07-19

**Authors:** Hui Liu, Caixia Zang, Junmei Shang, Zihong Zhang, Lu Wang, Hanyu Yang, Chanjuan Sheng, Fangyu Yuan, Cheng Ju, Fangyuan Li, Yang Yu, Xinsheng Yao, Xiuqi Bao, Dan Zhang

**Affiliations:** 1 State Key Laboratory of Bioactive Substrate and Function of Natural Medicine, Institute of Materia Medica, Chinese Academy of Medical Sciences and Peking Union Medical College, Beijing, China; 2 Institute of TCM & Natural Products College of Pharmacy, Jinan University, Guangzhou, China

**Keywords:** GJ-4, hyperlipidemia, vascular dementia, microglial polarization, PPAR-*γ*

## Abstract

**Background:**

GJ-4 is extracted from *Gardenia jasminoides* J. Ellis (Fructus Gardenia) with crocin composition and has been demonstrated to improve memory deficits in several dementia models in our previous studies.

**Objective:**

This study aimed to evaluate the effects of GJ-4 on hyperlipidemic vascular dementia (VD) and explore the underlying mechanisms.

**Design:**

In the current study, we employed a chronic hyperlipidemic VD rat model by permanent bilateral common carotid arteries occlusion (2-VO) based on high-fat diet (HFD), which is an ideal model to mimic the clinical pathogenesis of human VD.

**Results:**

Our results showed that GJ-4 could significantly reduce serum lipids level and improve cerebral blood flow in hyperlipidemic VD rats. Additionally, treatment with GJ-4 remarkedly ameliorated memory impairment and alleviated neuronal injury. Mechanistic investigation revealed that the neuroprotective effects of GJ-4 might be attributed to the inhibition of microglia-mediated neuro-inflammation via regulating the M1/M2 polarization. Our data further illustrated that GJ-4 could regulate the phenotype of microglia through activating the peroxisome proliferator-activated receptor-γ (PPAR-γ) and subsequently inhibited nuclear factor-κB (NF-κB) nuclear translocation and increased CCAAT/enhancer-binding protein β (C/EBPβ) expression.

**Conclusion:**

Our results implied that GJ-4 might be a promising drug to improve VD through the regulation of microglial M1/M2 polarization and the subsequent inhibition of neuro-inflammation.

**Figure:**
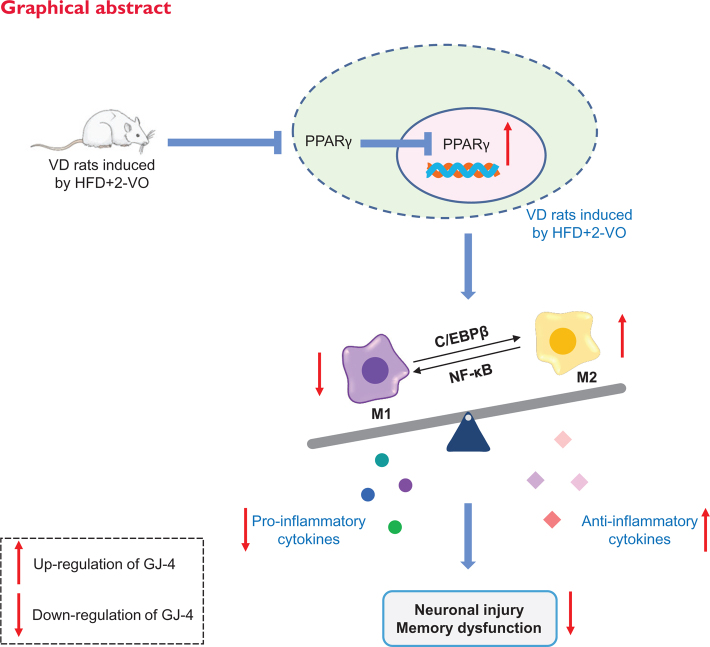


## Popular scientific summary

1. *Gardenia jasminoides* J. Ellis extract GJ-4 remarkedly improved dyslipidemia and memory disorders in hyperlipidemic vascular dementia (VD) rats.
2. The study showed that GJ-4 significantly inhibited microglial activation and modified microglial M1/M2 polarization by regulating PPAR-γ signaling pathway.
3. GJ-4 might be a promising drug to improve VD through the regulation of microglial M1/M2 polarization and the subsequent inhibition of neuro-inflammation.

Vascular dementia (VD) is currently recognized as the most frequent form of dementia second only to Alzheimer’s disease (AD) (1–3). VD patients often suffer from locomotor abnormalities, disorientation, forgetfulness, depression, and anxiety, as well as loss of capacities for problem solving and reasoning (2, 4). VD is resulted from accumulated damage in the vascular system, and traditional vascular risk factors (hyperlipidemia, hypertension, and diabetes) can also contribute to the pathogenesis of VD (5). Hyperlipidemia is an especially important factor, as it is thought to be related to the cerebral perfusion decline and breakdown of the blood–brain barrier (BBB). Both elevated low-density lipoprotein cholesterol (LDL-C) level and reduced high-density lipoprotein cholesterol (HDL-C) level are related to heightened risk of carotid atherosclerosis, leading to cognitive deficits after cerebral hypoperfusion or embolism (5, 6). The cerebral ischemia model based on hyperlipidemia is an ideal model to mimic the clinical pathological basis and has been extensively used in drug evaluation for VD.

Although vascular risk factors are implicated in the pathology and mechanisms underlying VD, there are accumulating evidences that neuroinflammation plays a critical part in the progression (7). In VD, chronic hypoperfusion and thromboembolic incidents result in decreased cerebral blood supply and hypoxia, which then trigger microglial activation (2, 8). The excessive activation of microglia can exacerbate the damage of neurogenesis, neuronal cell growth, and synaptogenesis and synaptic plasticity, causing neurodegeneration and cell death (9). Recent researches showed that the activated microglia could be categorized into M1 and M2 subtypes (10). M1 phenotypic cells mainly associated with the augmentation of inflammatory responses by releasing pro-inflammatory factors (11), which would trigger neuronal death, accelerate synaptic damage, and worsen memory impairment. By contrast, M2 phenotypic cells participate in anti-inflammatory responses relevant to the repair of brain injury following ischemic injury via upregulating anti-inflammatory mediators (12, 13). Therefore, targeting the balance of M1/M2 polarization to regulate neuroinflammation is beneficial for various neurodegenerative diseases (12, 14).

Multiple transcription factors participate in the regulation of microglial polarization, including the peroxisome proliferator-activated receptors-γ (PPAR-γ). PPAR-γ that belongs to the nuclear receptor superfamily is pivotal in regulating cellular glucose uptake, protecting against atherosclerosis and controlling immune reactions (15). Additionally, PPAR-γ is distributed broadly in central nervous system (CNS) and can protect neurons by attenuating inflammatory responses (16, 17), which makes it a potential target for CNS disorders (15, 18). Several studies demonstrated that PPAR-γ participated in inflammation control through modulation of microglial polarization (19). Regulating the PPAR-γ pathway is recognized as an attractive therapeutical strategy for many progressive neurological disorders (20, 21).

*Gardenia jasminoides* J. Ellis (Fructus Gardenia) is a potential traditional herb with versatile biological activities and has been traditionally applied to improve symptoms of cardiovascular (22) and nervous systems (23, 24). Modern pharmacological research revealed that *Gardenia jasminoides* J. Ellis extract exhibited anti-inflammatory activity and protective effects on ischemic brain injury (25) and neurodegenerative disorders (26). GJ-4 is a *Gardenia jasminoides* J. Ellis extract with crocin composition, and the metabolite could cross the BBB (27). In our previous researches, GJ-4 could notably ameliorate cognitive disorders in various AD models (28). Recently, we found GJ-4 exhibited protective effects on VD developed via focal cerebral ischemia/reperfusion injury (29). In this study, we developed a chronic hyperlipidemic VD model by bilateral common carotid arteries occlusion (2-VO) in hyperlipidemic rats to further investigate the therapeutical effects and the underlying mechanism of GJ-4 on VD.

## Materials and methods

### Extraction of GJ-4

**Fig. 1:**
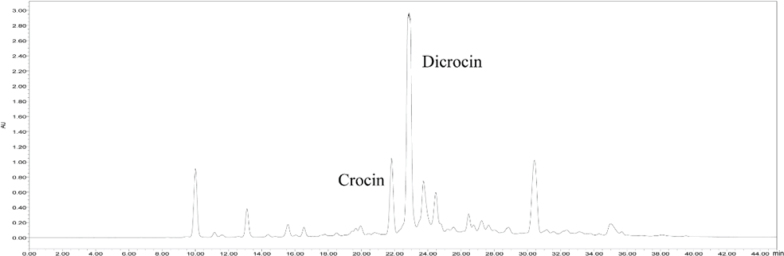
Major peaks in the crocin-rich fraction identified in HPLC-ELSD at 440 nm.

GJ-4 powder was prepared as previous description (30). The chromatographs are shown in Fig. 1.

### Experimental animals

Male Sprague–Dawley rats (160 to 180 g) were provided by Beijing Vital River Laboratory Animal Technology Co., Ltd. (Beijing, China) and fed in the diurnal lighting (12 h light/dark cycle) house. All experimental protocols were performed in accordance with the National Institutes of Health Guide for the Care and Use of Laboratory Animals and approved by the Animal Care and Use Committee of Peking Union Medical College.

### Development of hyperlipidemic VD rat model

Hyperlipidemic rats (*n* = 50) were developed by feeding with high-fat diet (HFD) for 7 weeks, while control rats (*n* = 10) with normal feeding. Thereafter, the 2-VO surgery was performed in the hyperlipidemic rats according to the methods described by Liu and Du with some modifications (31, 32). Briefly, after exposed and separated, each common carotid artery of HFD rats was ligated with a 5–0 type surgical silk suture. The sham-operated rats were subjected to the same operation except for arterial ligation.

### Treatment schedules

On the 2nd day after operation, the hyperlipidemic VD rats were randomly assigned into HFD+2-VO group, GJ-4 group (10 and 50 mg/kg, provided by Jinan University), and Ginkgo biloba extract group (EGb761, purchased from Dr. Willmar Schwabe). GJ-4 and EGb761 were orally administered one time per day for 3 weeks. Moreover, rats were maintained on their respective diets during treatment period.

### Step-down test

The apparatus consists of a square reflecting chamber and a cylindrical insulation platform. In this study, the step-down test was performed as previously described (29). After adapt for 2 min, rat would receive a foot shock immediately once it steps down from the platform during the next 3 min. Twenty-four hours after training, the rat was placed on the platform again. The following two parametric measures of retention were recorded as the final results in a blinded manner: the time to stay on the platform and the times jumping from the platform.

### Morris water maze test

The Morris water maze test was performed in a black cylindrical tank filled with water maintained at room temperature. The Morris water maze test was performed as previously described (28). During the orientation navigation test, a platform was underneath the water. A rat was randomly placed into the water at four equidistant locations for 4 days. If the rat can find platform and stayed for over 3 s, the latency will be recorded. If not, the latency will be 90 s, and the rat will be remained on the platform for another 30 s. The spatial probe test was carried out without the platform 24 h after the last orientation navigation test. Rats were placed in the pool to swim within 90 s, and the latency to cross the platform, time spent in the target quadrant, and the times of platform crossings were automatically recorded.

### Cerebral blood flow detection

Cerebral blood flow was measured by a laser doppler flowmetry. After the rats were anesthetized, a midline scalp incision was made to expose the skull bone. The probe of the laser doppler probe was fixed on the frontal brain to measure the brain blood supply. The mean blood flow of each rat was recorded.

### Blood lipid analysis

Blood samples from the caudal vein of rats were stayed 2 h and subsequently centrifuged at 3,500 rpm for 25 min. The levels of total cholesterol (TC), triglycerides (TG), LDL-C, and HDL-C were detected using commercial kits.

### Nissl staining

Briefly, after paraformaldehyde-fixed and paraffin-embedded, the brains were coronally sectioned to 3 mm thick sections. The slides were then subjected to dewaxing and rehydration, followed by stained with Nissl staining solution for 1 h at 50 °C. Following clearing and sealing, the images of Nissl-stained cells were obtained by the light microscope (NIKON E600, Japan).

### Immunohistochemistry analysis

Paraffin slices were incubated with CD11b antibody (Abcam, 1:200) and followed by biotinylated secondary antibody (Abcam, 1:2000). Hydrogen peroxidase and 3,3′-diaminobenzidine were used as chromogen to visualize the positive cells. Images of CD11b-positive cells were acquired by a light microscope (NIKON E600, Japan).

### Real-time polymerase chain reaction

The total mRNA expression of CD68, CD86, arginase 1 (Arg1), chitinase-like protein-3 (Ym1/Chi3l3), tissue necrosis factor-α (TNF-α), IL-1β, transforming growth factor-β (TGF-β), and IL-4 in the cortex and hippocampus was determined by real-time polymerase chain reaction (RT-PCR). Rats were anesthesia and rapidly decapitated, and cortex and hippocampus were rapidly isolated and stored at −80°C. Total RNA was extracted and reverse-transcribed by commercial kit (TransGen Biotech, China). The primer sequences for RT-PCR were listed in Table 1. The PCR was performed as previously described. The relative mRNA levels were analyzed by the 2^−∆∆Ct^ method normalizing to GAPDH and relative to the sham-operated groups.

**Table 1:** Primers for quantitative PCR

Gene	Primer sequence (5’-3’)	
	Forward	Reverse
CD68	ATGGTTCCCAGCCATGTGTT	**TTTCCACCCTGGGTCAGGTA**
CD86	GACACCCACGGGATCAATTA	**GCCTCCTCTATTTCAGGTTCAC**
Arg1	AAGAAAAGGCCGATTCACCT	**CACCTCCTCTGCTGTCTTCC**
Ym1	GATCACCACCCCTATGACCCT	**GGGACCAGTTGGTGTAGTAGC**
TNF-α	TCTCAAAACTCGAGTGACAAGC	**GGTTGTCTTTGAGATCCATGC**
IL-1β	TGATGTTCCCATTAGACAGC	**GAGGTGCTGATGTACCAGTT**
TGF-β	ACTCCCAACTACAGAAAAGCA	**GGTGGTGCCCTCTGAAATGA**
IL-4	TTGCTGTCACCCTGTTCTGC	**TTCTCCGTGGTGTTCCTTGTT**
GAPDH	AGTGCCAGCCTCGTCTCATA	**GGTAACCAGGCGTCCGATAC**

### Western blot

Rat tissues were lysed and then protein-quantified using a Bicinchoninic Acid (BCA) kit. Samples containing 40 μg proteins were separated via 10% sodium dodecyl sulfate polyacrylamide gel electrophoresis (SDS-PAGE) and subsequently transferred to polyvinylidene fluoride (PVDF) membranes. The membranes were then blocked with skim milk and incubated with primary antibodies: PPAR-γ, nuclear factor kappa beta (NF-κB), and CCAAT/enhancer-binding protein β (C/EBP β) (Abcam, 1:1000) at 4°C overnight, followed by corresponding secondary antibody (Abclonal, 1:2000) for 2 h at 37°*C*. The immunoreactive blots were visualized by LAS4000 software.

### Statistical analysis

All data were presented as mean ± SEM from at least three independent experiments. Statistical analysis was performed by one-way analysis of variance (ANOVA) followed by Tukey’s t-test. A *P* value of <0.05 was considered statistically significant.

## Results

### GJ-4 treatment restored dyslipidemia in hyperlipidemic VD rats

**Fig. 2:**
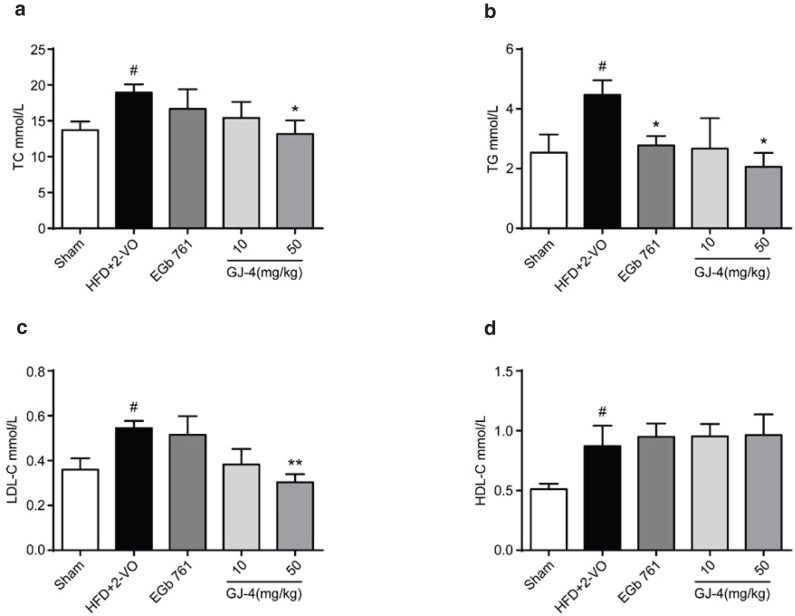
GJ-4 improved serum lipid abnormalities in HFD+2-VO-induced VD rats. After 3 weeks of GJ-4 administration, the effects of GJ-4 on lipid levels of VD rats were evaluated. (a) Serum TC concentrations. (b) Serum TG concentrations. (c) Serum LDL-C concentrations. (d) Serum HDL-C concentrations. The dosage of EGb761 was 50 mg/kg. Results were expressed as mean ± SEM from 8 to 10 rats. **^#^***P* < 0.05 versus sham-operated rats; *P < 0.05, **P < 0.01 versus HFD+2-VO rats.

The consumption of HFD can cause a leading obesity and related complications including hyperlipidemia (33). Before drug administration, rats fed with HFD for 7 weeks showed the significant increased TC, TG, and LDL-C levels (data not shown), indicating the successful establishment of hyperlipidemic model. Next, the 2-VO surgery was performed after 7 weeks of HFD, and then rats were administrated with GJ-4. After 3 weeks of GJ-4 (50 mg/kg) treatment, a remarkable decrease in serum TC, TG, and LDL-C levels was observed compared with the HFD+2-VO rats (TC: *P* < 0.05, TG: *P* < 0.05, and LDL-C: *P* < 0.01) (Fig. 2a–c). GJ-4 treatment had not shown significant effect on HDL-C level (Fig. 2d). The results suggested that GJ-4 significantly improved hyperlipidemia, which was induced by HFD.

### GJ-4 treatment alleviated memory decline and improved regional cerebral blood flow in hyperlipidemic VD rats

**Fig. 3:**
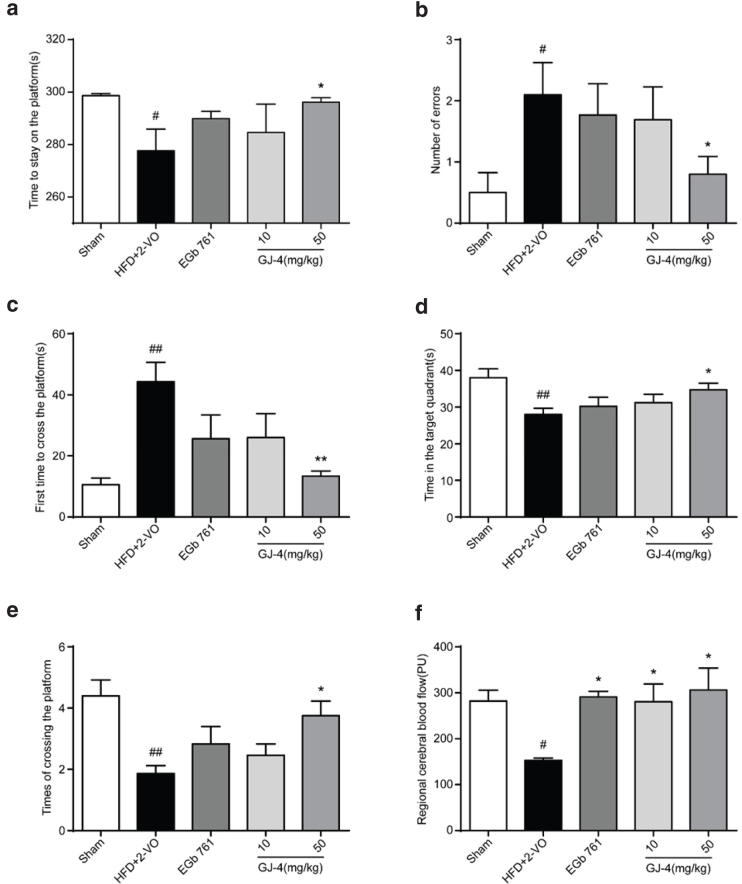
GJ-4 ameliorated memory decline and improved regional cerebral blood flow in HFD+2-VO-induced VD. The learning and memory ability of VD rats was accessed by the step-down test and Morris water maze test. (a) Time to stay on the platform in the step-down test. (b) Number of errors in the step-down test. (c) Time to first cross the platform in Morris water maze test. (d) Time in the target quadrant in Morris water maze test. (e) Number of crossing the platform in Morris water maze test. The cerebral blood flow of rats was detected by laser doppler flowmetry. (f) Regional cerebral blood flow of rats. The dosage of EGb761 was 50 mg/kg. Results were expressed as mean ± SEM from 10 to 12 rats. ^#^*P* < 0.05, ^##^*P* < 0.01 versus sham-operated rats; *P < 0.05, **P < 0.01 versus HFD+2-VO rats.

To evaluate the cognitive ability, we performed the step-down test on the 15th day, and Morris water maze experiment on the 17th day of GJ-4 administration. In the step-down test, the time of HFD+2-VO rat staying on the platform was obviously decreased (*P* < 0.05, Fig. 3a), and the number of errors was significantly increased (*P* < 0.05, Fig. 3b), suggesting that the animal subjected to HFD+2-VO exhibited severe memory impairment. GJ-4 at 50 mg/kg could apparently prolong the time staying on the platform and reduce the number of errors (Fig. 3a, b).

In the Morris water maze test, severe cognitive dysfunction was detected in HFD+2-VO rats, as indicated by delayed time to first cross the platform, decreased time spent in the target quadrant, and reduced number of platform crossing (Fig. 3c–e). Compared to the HFD+2-VO group, the rat administered with GJ-4 spent less time to first reach the platform (HFD+2-VO group, 44.33 s; GJ-4 50 mg/kg, 13.37 s) (Fig. 3c) and more time in the target quadrant (HFD+2-VO group, 28.03 s; GJ-4 50 mg/kg, 34.73 s) (Fig. 3d) and showed increased number of platform crossing (HFD+2-VO group, 1.84; GJ-4 50 mg/kg, 3.75) (Fig. 3e). The results illustrated that GJ-4 could remarkedly attenuate memory deficits in HFD+2-VO-induced VD rats.

The 2-VO operation results in the insufficiency of the persistent cerebral blood flow, which was partly similar to the clinical characteristics of VD patients (34, 35), so we then used laser doppler flowmetry to explore the effects on cerebral blood flow. As shown in Fig. 3f, regional cerebral blood flow was significantly decreased by 42% in HFD+2-VO rats. Administration of GJ-4 at both 10 and 50 mg/kg could improve the cerebral blood flow (HFD+2-VO group, 150.57 PU; GJ-4 10 mg/kg, 290.57 PU; GJ-4 50 mg/kg, 325.20 PU).

### GJ-4 attenuated neuronal injury of hyperlipidemic VD rats

**Fig. 4:**
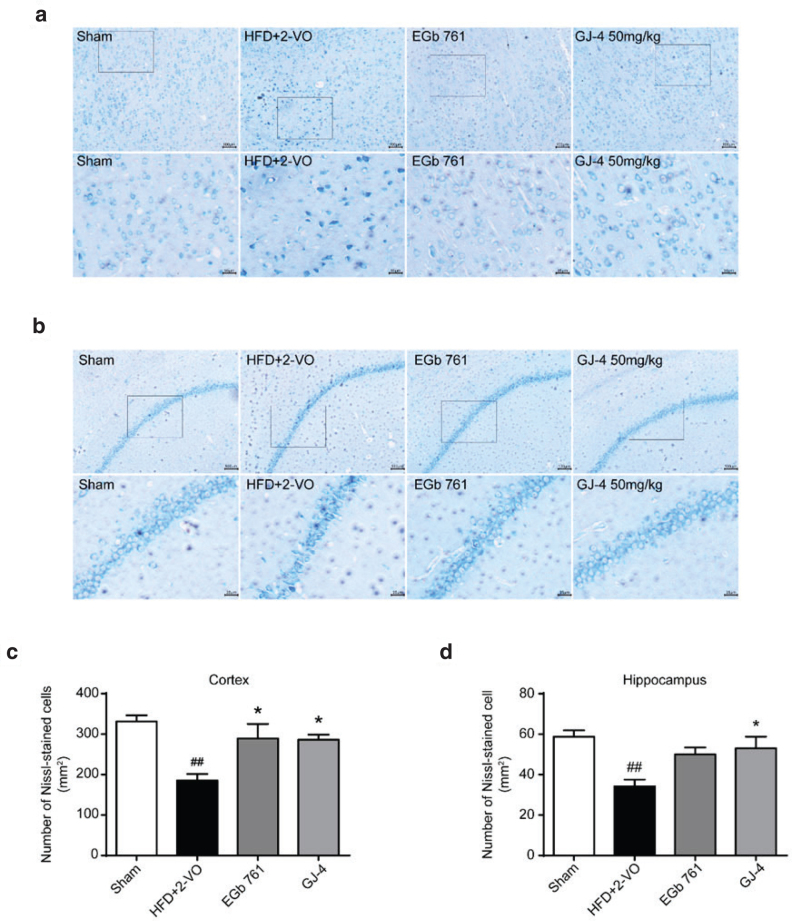
GJ-4 alleviated neuronal injury of HFD+2-VO-induced VD rats. Rats were fed with HFD for 7 weeks followed by 2-VO surgery, and then they were treated with GJ-4 for 3 weeks. (a) Nissl staining in the cortex. (b) Nissl staining in the CA1 region of hippocampus. (c) Statistical analysis of Nissl-stained cells in the cortex. (d) Statistical analysis of Nissl-stained cells in the CA1 region of hippocampus. The dosage of EGb761 and GJ-4 was both 50 mg/kg. Results were expressed as mean ± SEM from 3 to 5 rats. ^##^*P* < 0.01 versus sham-operated rats; *P < 0.05 versus HFD+2-VO rats.

Chronic cerebral ischemia initiates extensive neuronal loss and dysfunction, subsequently causing memory disorders. Nissl staining results revealed that the number of neurons in cortex and hippocampus CA1 region of HFD+2-VO rats was markedly decreased in comparison with the sham-operated rats (Cortex: sham-operated group, 331 cells/mm^2^; HFD+2-VO group, 185 cells/mm^2^. Hippocampus CA1: sham-operated group, 58.75 cells/mm^2^; HFD+2-VO group, 34.25 cells/mm^2^), detected by the Nissl staining. Moreover, HFD+2-VO rats presented large population of damaged neurons with vacuolated cytoplasm and pyknotic nucleus. To our expectation, GJ-4 treatment notably ameliorated neuronal injury of VD rats, as demonstrated by increased quantity and improved form (Fig. 4a–d). The above data indicated that GJ-4 ameliorated neuronal damage in VD rats challenged via hypoperfusion and hyperlipidemia.

### GJ-4 inhibited microglial activation and switched microglial phenotype in hyperlipidemic VD rats

**Fig. 5:**
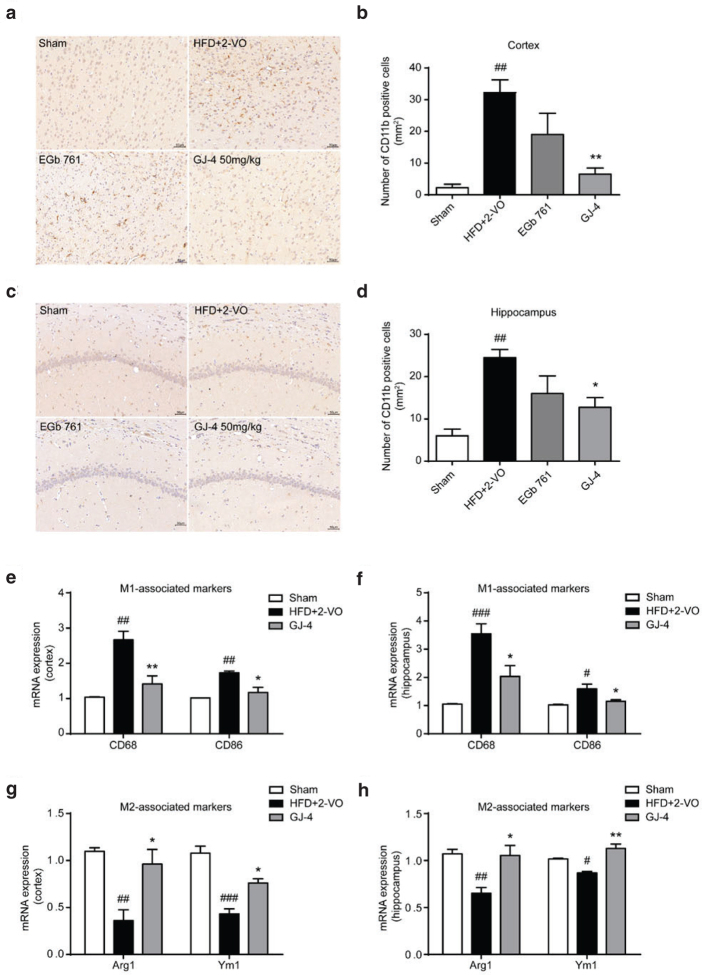
GJ-4 suppressed microglial activation and modulated microglial M1/M2 polarization in HFD+2-VO-induced VD rats. Rats were fed with HFD for 7 weeks followed by 2-VO surgery, and then they were treated with GJ-4 for 3 weeks. (a) CD11b staining in the cortex. (b) Statistical analysis of CD11b positive cells in the cortex. (c) CD11b staining in the hippocampus. (d) Statistical analysis of CD11b positive cells in the hippocampus. (e) CD68 and CD86 mRNA expressions in the cortex. (f) CD68 and CD86 mRNA expressions in the hippocampus. (g) Arg1 and Ym1 mRNA expressions in the cortex. (h) Arg1 and Ym1 mRNA expressions in the hippocampus. The dosage of EGb761 and GJ-4 was both 50 mg/kg. Results were shown as mean ± SEM from 3 to 5 rats. ^#^*P* < 0.05, ^##^*P* < 0.01, ^###^*P* < 0.001 versus sham-operated rats; *P < 0.05, **P < 0.01 versus HFD+2-VO rats.

A growing number of evidences indicated that neuroinflammation, mainly elicited by microglia, aggravated the pathological process of VD by exacerbated the production of pro-inflammatory cytokines. To identify whether the improved effects of GJ-4 on VD were concerned with the inhibition of neuroinflammation, we first examined the expression of CD11b, a typical marker of microglia. As shown in Fig. 5, the number of CD11b-positive cells in HFD+2-VO rats was obviously increased in comparison with the sham-operated rats. Besides, the microglia of HFD+2-VO rats were highly ramified, with enlarged cellular bodies and increased branches (Fig. 5a, c). Treatment of GJ-4 at 50 mg/kg markedly reduced the quantity of CD11b-positive cells (Cortex: HFD+2-VO group, 32.25 cells/mm^2^; GJ-4 50 mg/kg group, 6.5 cells/mm^2^. Hippocampus CA1: HFD+2-VO group, 24.5 cells/mm^2^; GJ-4 50 mg/kg group, 12.75 cells/mm^2^) (Fig. 5a–d), suggesting that GJ-4 could inhibit microglial activation in HFD+2-VO-induced VD rats.

Activated microglia is distinguished by the expression of specific phenotype markers. M1 phenotypic cells express typical phenotypic molecules, such as CD68 and CD86, while M2 phenotypic cells express Arg1 and Ym1. Our RT-PCR results showed that the levels of M1-polarizing markers (CD68 and CD86) were increased, whereas M2 marker expression levels (Arg1 and Ym1) were decreased in HFD+2-VO rats (Fig. 5e–h), suggesting that phenotypic changes occurred in microglia, ranging from anti-inflammatory M2 to pro-inflammatory M1 under hypoperfusion and hyperlipidemia conditions. Interestingly, GJ-4 50 mg/kg balanced the polarization condition of microglia by promoting the expression of M2 markers as well as inhibiting the expression of M1 markers (Fig. 5e–h). Altogether, our data indicated that protective effects of GJ-4 on VD induced by HFD+2-VO might be associated with microglial polarization regulation.

### GJ-4 regulated the secretion of inflammatory cytokines in hyperlipidemic VD rats

**Fig. 6:**
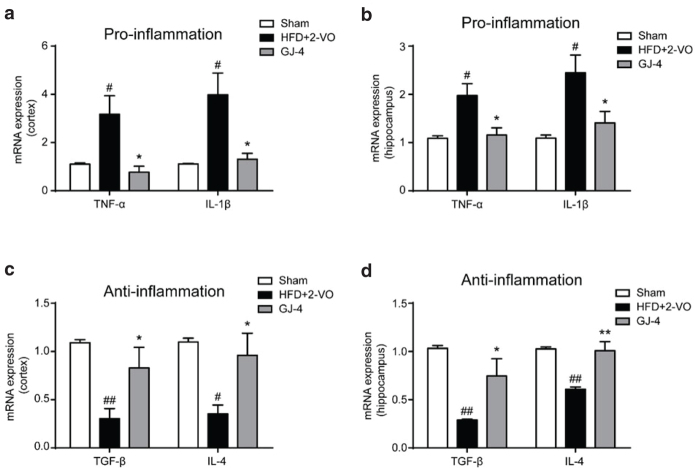
GJ-4 inhibited pro-inflammatory cytokine release and promoted anti-inflammatory cytokine production in HFD+2-VO-induced VD rats. Rats were fed with HFD for 7 weeks followed by 2-VO surgery, and then they were treated with GJ-4 for 3 weeks. (a) TNF-α and IL-1β mRNA expressions in the cortex. (b) TNF-α and IL-1β mRNA expressions in the hippocampus. (c) TGF-β and IL-4 mRNA expressions in the cortex. (d) TGF-β and IL-4 mRNA expressions in the hippocampus. The dosage of GJ-4 was 50 mg/kg. Results were shown as mean ± SEM from 4 to 5 rats. ^#^*P* < 0.05, ^##^*P* < 0.01 versus sham-operated rats; *P < 0.05, **P < 0.01 versus HFD+2-VO rats.

After the ischemic injury, microglia showed phenotypic transition overtime, switching from the beneficial M2 into the harmful M1 type (14). In this study, levels of TNF-α and IL-1β, representative of M1-related cytokines, were significantly increased in HFD+2-VO-induced VD rats (Cortex: TNF-α, *P* < 0.05; IL-1β, *P* < 0.05. Hippocampus: TNF-α, *P* < 0.05; IL-1β, *P* < 0.05) (Fig. 6a, b). Additionally, a significant reduction of the M2-associated cytokines (TGF-β and IL-4) was also found in HFD+2-VO rats (Cortex: TGF-β, *P* < 0.01; IL-4, *P* < 0.05. Hippocampus: TGF-β, *P* < 0.01; IL-4, *P* < 0.01) (Fig. 6c, d). GJ-4 treatment could markedly decrease the mRNA expression of TNF-α and IL-1β, while the levels of TGF-β and IL-4 mRNA were increased by treatment with GJ-4 in VD rats (Fig. 6a–d), indicating that GJ-4 could inhibit microglial M1 polarization and promote M2 polarization. Taken together, the above results further certified that GJ-4 might suppress neuroinflammation through promoting the phenotypic shift of microglia from M1 to M2 phenotype in hyperlipidemic VD rats.

### GJ-4 increased the activity of PPAR-γ in hyperlipidemic VD rats

**Fig. 7:**
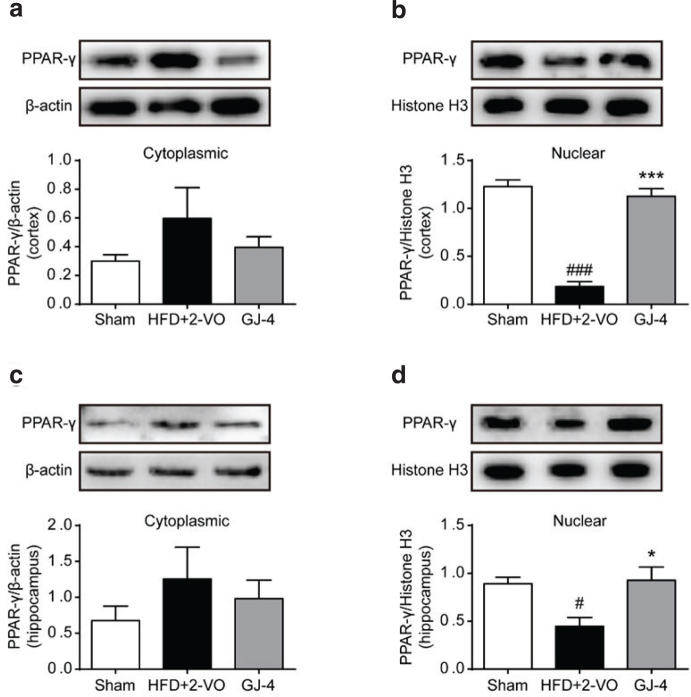
GJ-4 stimulated PPAR-γ nuclear translocation. Rats were fed with HFD for 7 weeks followed by 2-VO surgery, and then they were treated with GJ-4 for 3 weeks. (a) PPAR-γ expression in the cytoplasm of cortex. (b) PPAR-γ expression in the nucleus of cortex. (c) PPAR-γ expression in the cytoplasm of hippocampus. (d) PPAR-γ expression in the nucleus of hippocampus. The dosage of GJ-4 was 50 mg/kg. Results were shown as mean ± SEM from 4 to 5 rats. ^#^*P* < 0.05, ^###^*P* < 0.001 versus sham-operated rats; *P < 0.05, ***P < 0.001 versus HFD+2-VO rats.

PPAR-γ stimulation is considered necessary for inhibiting inflammatory response (36) and is implied in controlling the microglial alternative activation and regulating microglial M1/M2 polarization (19, 37). To further explore the therapeutic mechanism of GJ-4 on VD, Western blot was employed to test the profiles of PPAR-γ expression in VD rats. As shown in Fig. 7, the levels of PPAR-γ were elevated in cytoplasm but were significantly reduced in nuclei, indicating that HFD+2-VO inhibited the nuclear translocation of PPAR-γ. Treatment with GJ-4 apparently promoted PPAR-γ nuclear translocation (Fig. 7a–d), as demonstrated by upregulated PPAR-γ expression in nuclei and downregulated expression in cytoplasm. The earlier data suggested that GJ-4 could regulate microglial M1/M2 polarization via promoting PPAR-γ activation.

### GJ-4 regulated PPAR-γ-induced microglial polarization via NF-κB and C/EBPβ in hyperlipidemic VD rats

**Fig. 8:**
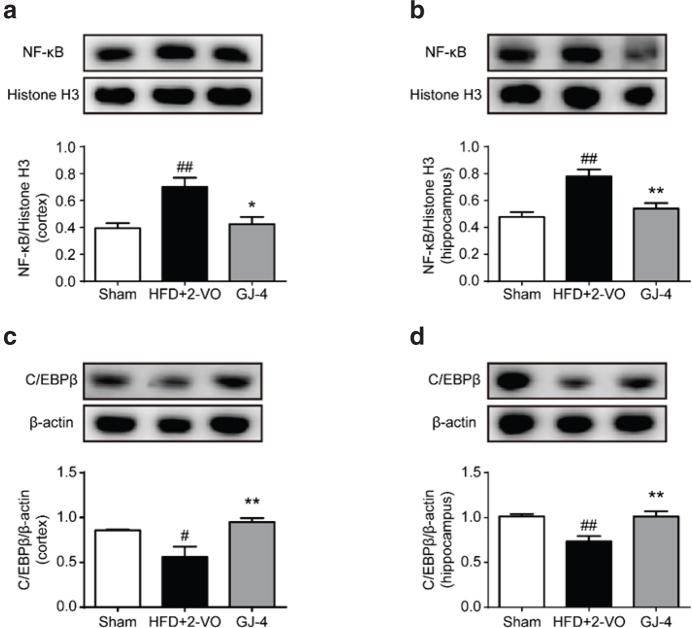
GJ-4 suppressed NF-κB nuclear translocation and elevated C/EBPβ expression in HFD+2-VO-induced VD rats. Rats were fed with HFD for 7 weeks followed by 2-VO surgery, and then they were treated with GJ-4 for 3 weeks. (a) NF-κB expression in the nucleus of cortex. (b) NF-κB expression in the nucleus of hippocampus. (c) C/EBPβ expression in the cortex. (d) C/EBPβ expression in the hippocampus. The dosage of GJ-4 was 50 mg/kg. Results were shown as mean ± SEM from 4 to 5 rats. ^#^*P* < 0.05, ^##^*P* < 0.01, ^###^*P* < 0.001 versus sham-operated rats; *P < 0.05, **P < 0.01 versus HFD+2-VO rats.

Recent studies have shown that the PPAR-γ activation was potential to modulate microglia-mediated neuroinflammation through the regulation of different signaling pathways, such as the NF-κB and the C/EBPβ pathways (38). NF-κB plays an essential role in the shift from the anti-inflammatory M2 to the pro-inflammatory M1 phenotypes and then increases the expression of relevant pro-inflammatory cytokines through translocating into nucleus (39, 40). In this study, the nuclear translocation of NF-κB was apparently increased in rats subjected to HFD+2-VO (Fig. 8a, b), indicating the excessive activation of NF-κB pathway. GJ-4 markedly suppressed the activity of NF-κB by inhibiting its nuclear translocation (Fig. 8a, b). C/EBPβ can specifically shift the microglia toward an anti-inflammatory phenotype (41). Western blot analysis revealed that the C/EBPβ expression was significantly reduced in VD rats. GJ-4 treatment obviously increased the C/EBPβ expression compared with the VD rats (Fig. 8c, d), indicating that the C/EBPβ pathway was activated by GJ-4. The above results revealed that both decreased M1-polarization and increased M2-polarization by GJ-4 on VD rats upon PPAR-γ activation might be regulated by NF-κB and C/EBPβ pathways.

## Discussion

In the present study, through employing the hyperlipidemic VD model developed via 2-VO in HFD-fed rats, we found that treatment with GJ-4 could significantly restore the abnormal serum lipid levels, increase cerebral blood flow, protect neurons, and improve memory ability. Further investigation revealed that GJ-4 could modulate microglial M1/M2 polarization by suppressing M1 phenotype and promoting M2 phenotype via regulating the activation of the PPAR-γ.

There are a variety of pathological mechanisms implied in the development of VD, and lipid is attracting more and more attention. Growing evidences have proved increased levels of TC, TG, and LDL-C as well as reduced levels of HDL-C are known as risk factors for carotid atherosclerosis (5), which may result in cerebral hypoperfusion or embolism and subsequent cognitive dysfunction (6). Thus, in this study, we developed a hypoperfusion-induced VD model in hyperlipidemic rats via 2-VO surgery. The model rats were characterized by dyslipidemia, chronic cerebral ischemia, and cognitive impairment, which conformed to the clinical pathological basis and was similar with memory deficits of human beings (42). In accordance with the reported studies, we found that HFD+2-VO rats developed hyperlipidemia as shown by higher levels of TC, TG, and LDL-C and reduced cerebral blood supply as well as severe memory deficits compared with the sham-operated rats (42). In this hyperlipidemic VD model, GJ-4 showed the marked effect on improving memory functions and certain effect on reducing plasma lipids. Memory function was closely associated with the serum lipid state, which implied us that further investigation was needed to clarify the effects of GJ-4 on improving memory.

Microglia are the major immune cellular in CNS, and accumulating researches suggested that microglia-mediated neuroinflammation participates in the pathogenesis and progression in various neurodegenerative diseases, such as AD and Parkinson’s disease (43–45). Many evidences point to the neurotoxic effect of excessive microglial activation, and modifying microglial activation might be a beneficial strategy for various neurodegenerative disorders (45, 46). However, recent research found that simply blocking inflammation by suppressing microglial activation would likely not induce overall beneficial effects (43), and increasing studies focused on the microglial polarization regulation. Similar to periphery macrophages, microglia are heterogeneous with different phenotypes and functions in response to microenvironmental disturbances, such as chronic cerebral ischemia (47), which range from pro-inflammatory M1 phenotype to anti-inflammatory M2 phenotype (14). M1 phenotype microglia could synthesize and secrete pro-inflammatory cytokines, aggravating neuronal injury and CNS disorders. On the contrary, M2 phenotype participates in the repair process after brain injury by removing damaged cell debris and generating neurotrophic factors and anti-inflammatory mediators (48). It has been reported that restoring the M1/M2 balance of microglia could reverse memory decline in AD mice (49) and attenuate brain damage of the focal cerebral ischemia model (47). Thus, redirection of microglial M1/M2 status by inhibiting the M1 phenotype while stimulating the M2 phenotype has been suggested as an effective strategy for neurodegenerative diseases (50, 51). In this VD rat model, we found the occurrence of microglial over-activation and abnormal microglial phenotypic transformation. Treatment with GJ-4 notably suppressed microglial activation by switching the phenotypes of microglia, as evidenced by decreased mRNA levels of M1-associated molecules as well as increased mRNA levels of M2-associated molecules. These data identified that protective effects of GJ-4 were on the account of suppressing microglia-mediated neuroinflammatory actions via regulating microglial polarization.

Recently, PPAR-γ is considered as the potential therapeutic target for various CNS diseases (52). It has been reported that PPAR-γ activation could elicit potent neuroprotective effects in various animal models of cerebral ischemia, AD, and VD (21, 53–55). The neuroprotective effects of PPAR-γ are closely involved in the regulation of microglia-associated neuroinflammation (56). Exactly, the activation of PPAR-γ could suppress microglial M1 phenotype polarization via inhibiting the activation of NF-κB and could also cooperate with C/EBPβ to subsequently promote M2 microglial polarization (37, 57). To further investigate the mechanisms of GJ-4 in regulating microglia-mediated neuroinflammation, we analyzed the changes of NF-κB and C/EBPβ. The PPAR-γ nuclear translocation in VD rats was decreased, which consequently caused downstream increased activity of NF-κB as well as decreased expression of C/EBPβ. GJ-4 markedly increased the activity of PPAR-γ by promoting its nuclear translocation, and further investigation demonstrated that GJ-4 could inhibit NF-κB nuclear translocation and elevate the C/EBPβ expression. These aforementioned data suggested that with the activation of PPAR-γ, GJ-4 switched the microglial phenotypes via modulating NF-κB and C/EBPβ signaling pathways.

## Conclusions

In summary, our study revealed that GJ-4 could improve dyslipidemia and memory impairment in hyperlipidemic VD rats. The protective effects may be related to its ability to regulate microglial polarization. Mechanistic studies showed that GJ-4 could switch the microglial phenotype through the activation of PPAR-γ signaling pathway. Collectively, our data supported that GJ-4 might become an effective alternative for VD treatment.
